# Sensitive Tumorigenic Potential Evaluation of Adult Human Multipotent Neural Cells Immortalized by hTERT Gene Transduction

**DOI:** 10.1371/journal.pone.0158639

**Published:** 2016-07-08

**Authors:** Kee Hang Lee, Hyun Nam, Da Eun Jeong, Sung Soo Kim, Hye Jin Song, Hee Jang Pyeon, Kyeongjin Kang, Seung-Cheol Hong, Do-Hyun Nam, Kyeung Min Joo

**Affiliations:** 1 Department of Health Sciences and Technology, SAIHST, Sungkyunkwan University, Seoul, South Korea; 2 Department of Neurosurgery, Samsung Medical Center, Sungkyunkwan University, Seoul, South Korea; 3 Department of Anatomy and Cell Biology, Sungkyunkwan University School of Medicine, Seoul, South Korea; 4 Stem Cell and Regenerative Medicine Center, Research Institute for Future Medicine, Samsung Medical Center, Seoul, 06351, South Korea; Swedish Neuroscience Institute, UNITED STATES

## Abstract

Stem cells and therapeutic genes are emerging as a new therapeutic approach to treat various neurodegenerative diseases with few effective treatment options. However, potential formation of tumors by stem cells has hampered their clinical application. Moreover, adequate preclinical platforms to precisely test tumorigenic potential of stem cells are controversial. In this study, we compared the sensitivity of various animal models for *in vivo* stem cell tumorigenicity testing to identify the most sensitive platform. Then, tumorigenic potential of adult human multipotent neural cells (ahMNCs) immortalized by the human telomerase reverse transcriptase (hTERT) gene was examined as a stem cell model with therapeutic genes. When human glioblastoma (GBM) cells were injected into adult (4–6-week-old) Balb/c-nu, adult NOD/SCID, adult NOG, or neonate (1–2-week-old) NOG mice, the neonate NOG mice showed significantly faster tumorigenesis than that of the other groups regardless of intracranial or subcutaneous injection route. Two kinds of ahMNCs (682TL and 779TL) were primary cultured from surgical samples of patients with temporal lobe epilepsy. Although the ahMNCs were immortalized by lentiviral hTERT gene delivery (hTERT-682TL and hTERT-779TL), they did not form any detectable masses, even in the most sensitive neonate NOG mouse platform. Moreover, the hTERT-ahMNCs had no gross chromosomal abnormalities on a karyotype analysis. Taken together, our data suggest that neonate NOG mice could be a sensitive animal platform to test tumorigenic potential of stem cell therapeutics and that ahMNCs could be a genetically stable stem cell source with little tumorigenic activity to develop regenerative treatments for neurodegenerative diseases.

## Introduction

The common pathological feature of various neurodegenerative diseases is progressive and irreversible tissue damage in the central nervous system (CNS) [1, 2]. The damage rarely recovers spontaneously, as the CNS has very limited regenerative potential [3]. Accordingly, current therapeutic methods to treat neurodegenerative diseases focus on delaying disease progression [1, 2]. To overcome this situation, neuro-regeneration with stem cells is emerging as a promising treatment because stem cells have differentiation potential into various cells to replace damaged neural tissue [1, 2]. Stem cell sources, such as bone marrow, adipose tissue, teeth, and placenta, can make neural cells [4–7]. However, their differentiation capacities remain controversial [8].

Although the underlying stem cell therapy treatment mechanism is differentiation into functional neural cells, paracrine factors secreted by stem cells also play important roles protecting damaged neural cells and stimulating endogenous neural stem cells [9, 10]. Stem cells have been genetically modified to secrete neurotrophic factors, such as brain-derived neurotrophic factor (BDNF) [11], glial cell line-derived neurotrophic factor (GDNF) [12], and nerve growth factor (NGF) [13, 14]. Adult human multipotent neural cells (ahMNCs) derived from the temporal lobe [7] could be a good candidate for genetic manipulation, as they harbor differentiation potential into neurons and astrocytes *in vitro* and *in vivo* and show significant therapeutic effects against stroke.

Genetic modification to stably express neurotrophic factors is usually accompanied by integration of genes into the host genome, which can disrupt the genome. Accordingly, biosafety issues of genetically engineered stem cells should be verified by proper methods from the preclinical stage. In this study, we investigated highly sensitive animal models to examine tumorigenicity of cells adequately, and then *in vivo* tumorigenic potential of genetically engineered ahMNCs was tested. AhMNCs immortalized by transferring the lentiviral human telomerase reverse transcriptase (hTERT)-gene (hTERT-ahMNCs) were utilized, as immortalization is the initial step of tumor formation [15]. Although immortalization by hTERT does not transform stem cells directly [15], it could increase the chances for of *in vivo* tumorigenesis more than transferring other therapeutic genes. Moreover, hTERT could alter functional characteristics of stem cells such as differentiation [16, 17]. The results of this study can be used to develop a sensitive protocol to examine *in vivo* tumorigenicity of genetically modified stem cells for clinical purposes. Moreover, genetic stability and low *in vivo* tumorigenic potential of ahMNCs will make them an adequate platform for genetically modified stem cell therapeutics to treat various neurodegenerative diseases.

## Materials and Methods

### Primary isolation and ahMNCs culture

Informed written consent was obtained from each patient according to the guidelines approved by the Samsung Medical Center Institutional Review Board (IRB File No. SMC 2009-07-071-004). A portion of the temporal lobe cerebral cortex was surgically removed from a patient with epilepsy. The two surgical specimens represented as 682TL (22-year-old male with hippocampal sclerosis in temporal lobe) and 779TL (26-year-old female with hippocampal sclerosis in temporal lobe) were derived from each patient, mechanically minced and then incubated in an enzyme solution containing 10 units/mL papain (Sigma, St. Louis, MO, USA), 0.1 mg/mL DNase I (Roche, Basel, Switzerland), and 4 mg/mL D, L-cysteine (Sigma) for 30 minutes at 37°C. After mild trituration, the cells were filtered through a 70-μm cell strainer (BD Biosciences, Franklin Lakes, NJ, USA). The filtered cells were purified with 30% Percoll (Sigma) by ultra-centrifugation at 20,000 rpm for 20 minutes at 18°C. The total number of cells was counted after a wash in PBS. The total numbers of isolated 682TL and 779TL cells were approximately 2.9 × 10^6^ and 4.4 × 10^6^, respectively. The cells were cultured on poly-L-ornithine (Sigma)-coated dishes with Dulbecco's Modified Eagle Medium: Nutrient Mixture F-12 (DMEM/F12) (Gibco, Paisley, Scotland, UK) media with the B27 supplement (Gibco, Grand Island, NY, USA), 1% penicillin/streptomycin cocktail (Gibco), 100 ng/mL epidermal growth factor (EGF) (R&D Systems, Minneapolis, MN, USA), 100 ng/mL basic fibroblast growth factor (bFGF) (R&D Systems), and 0.5% fetal bovine serum (FBS) (Gibco). Half of the culture media was changed with new culture media containing these supplements every 3 days. Sub-culturing was performed at 70~80% confluency using accutase (PAA Laboratories, Pasching, Austria).

### *In vivo* tumorigenicity animal model

This study was reviewed and approved by the Institutional Animal Care and Use Committee (IACUC) of Samsung Biomedical Research Institute (SBRI, Seoul, Korea) under approval number 20140916001. SBRI is an Association for Assessment and Accreditation of Laboratory Animal Care International (AAALAC International) accredited facility and abide by the Institute of Laboratory Animal Resources (ILAR) guide. Animal experiments were conducted in accordance with the Institute for Laboratory Animal Research Guide for the Care and Use of Laboratory Animals and followed protocols approved by the IRB at the Samsung Medical Center (Seoul, Korea). Balb-c/nu, NOD/SCID and NOG (IL2 receptor γ-chain truncated NOD/SCID) mice were utilized (Orient Bio., Seongnam, Korea). Adult male mice were anesthetized by intraperitoneal injection of 30 mg/kg Zoletil (Virbac Korea, Seoul, South Korea) and 10 mg/kg Rompun (Bayer Korea, Seoul, South Korea) before intracranial injection. Neonate male mice were anesthetized by 2~4% isoflurane (Hana Pharm. Co., Ltd., Hwaseong, South Korea) with oxygen until cessation of movement before intracranial injection. Subsequently, harvested cells were stereotactically (1.7 mm left and 0.5 mm anterior from the bregma, 3.3 mm in depth from the skull,) injected to adult mice using 28 G needle connected to infusion pump (KD Scientific Inc., Holliston, MA, USA) into the left striatum (2 × 10^5^ cells in 5 μL HBSS at 1 μL/min into each mouse, n = 10/group). In case of intracranial injection to neonatal mice, harvested cells were manually injected into the approximately left striatum (1.5 mm left and 1.0 mm anterior from the bregma, 3.0 mm in depth from the skin, 2 × 10^5^ cells in 5 μL HBSS into each mouse, n = 10/group) using 28 G syringe. Next, harvested cells were manually injected into the right flank subcutaneous using a 26 G syringe (2 × 10^6^ cells in 100 μL HBSS into each mouse, n = 10/group). All subcutaneous injection was performed under restraint without anesthesia. Tumor volume was estimated by the formula (tumor volume [mm^3^] = width [mm] × width [mm] × length [mm] × 0.5) when we measure at that time. After injection, all transplanted animals recovered on heating pads until they moved. And then, adult animals were returned to cages where they lived and neonatal animals were returned to their mothers. Finally, we measured body weight and tumor volume of animals every 3 days until they reached endpoint; 1) body weight reduction of more than 20% to compared with the highest body weight or 2) > 1,500 mm^3^ of tumor volume. Above mentioned animals were euthanized using CO_2_.

### Immunohistochemistry

The paraffin blocks were sectioned on a microtome (Leica, Wetzlar, Germany) at 4-μm thickness, and the sections were put on silane-coated microscope slides (Muto pure Chemicals Co., Ltd., Tokyo, Japan). The slides were heated on a slide warmer (Lab-line Instruments USA, Dubuque, IA, USA) for 35 minutes at 65°C. The sections were deparaffinized and rehydrated. The antigen was retrieved by heating the sections in a target retrieval solution (Dako, Carpentaria, CA, USA) at 125°C for 30 minutes. After cooling to room temperature, the slides were incubated in 3% hydrogen peroxide in methanol for 12 minutes to quench endogenous peroxidase. The following primary antibodies incubated at 4°C overnight; STEM121™ (1:500, StemCells, Inc., Newark, CA, USA) and anti-Ki-67 antibody (1:50, Abcam, Cambridge, UK). The slides were then washed three times in PBS and exposed to biotinylated secondary antibody for 1 hour and then to the avidin-biotin complex (Vector Laboratories Inc., Burlingame, CA, USA) for 1 hour. The slides were stained with DAB (3,3’-diaminobenzidine tetrahydrochloride), counterstained with hematoxylin, and scanned using the Scanscope AT (Aperio Technologies Inc., Visa, CA, USA).

### Lentivirus production and ahMNCs transduction

hTERT-overexpressing lentiviral vector was generously provided by Dr. Matthias Schieker [18]. The hTERT-overexpressing lentiviral vector was co-transfected with the VSVG and PAX2 lentiviral packaging vectors into HEK293FT cells (Invitrogen, Carlsbad, CA, USA). And then, the supernatants were collected on days 2, 3, and 4. The lentiviruses were concentrated by ultra-centrifugation. The concentrated lentiviruses were resuspended in 1 mL of PBS. For transduction of 682TL and 779TL, cells were seeded in 35mm-dish and cultured to be 70% confluency. The lentiviruses were added into the culture medium in the presence of polybrene (8 μg/mL) once. After 24 hours, cells were cultured with blasticidin (5 μg/mL) and medium was changed every other day. After 12 days of selection, proliferation rate and original morphology represented as hTERT-682TL and hTERT-779TL were validated for hTERT-682TL and hTERT-779TL cells. hTERT-682TL and hTERT-779TL were expanded serial passaging.

### *In vitro* differentiation of ahMNCs and hTERT-ahMNCs

Differentiation assay was conducted following previous report [7] with a little modification. Cells were cultured to be 80% confluent in 96-well plate and the medium was replaced by differentiation medium which was consisted of DMEM/F12, 0.5% FBS, B27, 0.5 mM 3-isobutyl-1-methylxanthine (IBMX) (Sigma), and 1% penicillin/streptomycin cocktail. Fresh differentiation medium was added every other day until more differentiated morphology was observed.

### Immunocytochemistry

Cells were fixed with ice-cold 4% paraformaldehyde (Biosesang, Gyeonggi, Korea) for 20 minutes at room temperature (RT) and permeabilized with 1% Triton X-100 (Sigma) for 5 minutes at RT. After washing with PBS, the cells were treated in a blocking solution containing PBS supplemented with 0.1% Triton X-100 (Sigma), 5% normal goat serum (Vector, Olean, NY, USA), and 5% normal horse serum (Vector) for 1 hour at RT. Then, the cells were incubated with primary antibodies overnight at 4°C; Nestin (1:2,500, Novus Biologicals, Littleton, CO, USA), STEM121™ (1:500, StemCells, Inc.), MAP-2 (1:200, Santa Cruz, Dallas, TX, USA), GFAP (1:2,000, Abcam). Goat anti-Mouse IgG (H+L) Secondary Antibody, Alexa Fluor® 488 conjugate (1:500, Thermo Fisher Scientific), Goat anti-Rabbit IgG (H+L) Secondary Antibody, Alexa Fluor® 488 conjugate (1:500, Thermo Fisher Scientific), and Goat anti-Chicken IgG (H+L) Secondary Antibody [DyLight 488] (1:500, Novus Biologicals) were used as secondary antibodies for 2 hours at RT. The cells were treated with DAPI (1:1,000, Thermo Fisher Scientific) for 5 minutes at RT. After washing with PBS, the slides were mounted with Vectashield™ (Vector) and analyzed using an Axio observer Z1 as a fluorescence microscope (Zeiss, Jena, Germany).

### Detection of hTERT by reverse transcription-polymerase chain reaction (RT-PCR)

Total RNA was obtained from ahMNCs and hTERT-ahMNCs using an RNeasy Mini Kit (Qiagen, Valencia, CA, USA). Total RNA (2 μg) was reverse-transcribed with using a first-strand cDNA synthesis kit (Life Technologies) following the manufacturer’s instructions. The resulting cDNA was used as the PCR template. PCR was performed with i-MAXII (Intron, Sungnam, Korea). The oligonucleotide sequences for hTERT were: (forward: 5'-GGAGCAAGTTGCAAAGCATTG-3'; reverse: 5'-TCCCACGACGTAGTCCATGTT-3') and β-actin (forward: 5’- ATGTGCAAGGCCGGCTTC-3’; reverse: 5’-ACCCATGCCCACCATCAC-3’). Reaction conditions were modification at 95°C for 2 minutes, denaturation at 95°C for 20 seconds, annealing at 57°C for 40 seconds, and extension at 72°C for 40 seconds. This cycle was repeated 35 times (or 25 times) and finished with an extension at 72°C for 5 minutes. The reaction products were separated by agarose gel electrophoresis with ethidium bromide.

### Relative telomere length measurements

Telomere length of ahMNCs and hTERT-immortalized ahMNCs was analyzed using specific primers for telomere and single copy 36B4 gene (encodes acidic ribosomal phosphoprotein) according to previous study [19]. Relative telomere length was determined by calculating T/S values using the formula T/S = 2^-∆Ct^, where ∆Ct = average Ct^telomere^—average Ct^36B4^. PCR reactions were performed in triplicate 20 μL reaction volumes (30 ng DNA sample per reaction) for all samples. The PCR mixture contained 5× HOT FIREPol® EvaGreen® qPCR Mix Plus (ROX) (Solis BioDyne, Tartu, Estonia) and 10 pM of each of the primers. The following primers were used: telomere (forward: 5’-GGTTTTTGAGGGTGAGGGTGAGGGTGAGGGTGAGGGT-3’; reverse: 5’-TCCCGACTATCCCTATCCCTATCCCTATCCCTATCCCTA-3) and 36B4 (forward: 5’-CAGCAAGTGGGAAGGTGTAATCC-3’; reverse: 5’-CCCATTCTATCATCAACGGGTACAA-3’). The PCR conditions were initial denaturation of 5 minutes at 95°C, followed by 40 cycles at 95°C for 5 seconds, 56°C for 30 seconds, and 72°C for 30 seconds using the Applied Biosystems 7500 Real-Time PCR System (Applied Biosystems, Foster City, CA, USA). All samples were run in triplicate, and the T/S ratio was calculated for each sample.

### Karyotype analysis

A colcemid (Gibco) stock solution was added to culture dishes containing ahMNCs and hTERT-ahMNCs and returned to the incubator (37°C, 5% CO_2_) for 4 hours. Subsequently, the treated cells were collected in tubes with 0.5% Trypsin-EDTA (Gibco) and centrifuged at 1,000 rpm for 10 minutes. After removing the supernatant, 5 mL of hypotonic solution (0.075 M KCl) was added to stand at 37°C for 10 minutes, followed by centrifugation at 1,000 rpm for 10 minutes. After aspirating the hypotonic solution carefully, 3 mL of Canoy’s fixative (methanol: acetic acid = 3:1) was added to the tube and mixed. After more than 20 minutes, the solution was centrifuged at 1,000 rpm for 10 minutes. The Canoy’s fixative was removed, and fresh fixative was added and aspirated one more time. The supernatant was discarded, leaving about 2 times Canoy’s fixative of pellets. The pellets were spread on prepared glass slides and warm 60°C for 30 minutes. The slides were treated with 50% H_2_O_2_ for 3 minutes. The slides were warmed at 60°C for 30 minutes and stained with G-banding technique. In all cases, 20 cells per sample were analyzed karyotypes using ChIPS-Karyo (Chromosome Image Processing System) (GenDix, Inc. Seoul, Korea).

### FACS analysis

For FACS analysis, cells were harvested and washed. Cells were fixed and permeabilized with a human neural lineage analysis kit (BD Biosciences) according to the manufacturer’s instruction. 1 × 10^5^ cells were stained with Alexa 488 (for MAP2 and Tuj1) or 647-conjugated (for Nestin and GFAP) antibodies against MAP2, Tuj1, Nestin, and GFAP (BD Biosciences) on ice for 30 minutes. The fluorescence intensity was measured on a FACSCalibur™ (BD, San Joes, CA, USA), and the data were analyzed using FLOWJO software (Tree Star, Inc., Ashland, OR, USA).

### Soft Agar Assay

Pre-warmed 0.8% bottom agar (Difco™ Agar) (BD, Franklin Lakes, NJ, USA) was prepared with PBS. 1 mL of bottom agar was poured into each well in 12-well plate. Pre-warmed 0.4% top agar (BD) was prepared with culture medium for each cell type. After bottom agar was congealed, 1mL of 0.4% top agar (BD) containing 500 cells of U87MG or 1,000 cells of ahMNCs or hTERT-ahMNCs was poured on the bottom agar. After top agar is congealed, wells were incubated at 37°C and 5% CO_2_ for 2 weeks. The medium was changed every 3 days.

### Statistics

Data were compared using Student’s *t*-test. All statistical analyses were two-tailed, and *p*-values < 0.05 were considered significant. All statistics were calculated using either Microsoft Office Standard 2013 (Microsoft, Redmond, WA, USA) or GraphPad PRISM 5 for Windows (GraphPad Software, Inc., San Diego, CA, USA).

## Results

### Sensitivity of the animal models for *in vivo* tumorigenicity testing

The sensitivities of various animal models for *in vivo* tumorigenicity test were compared to adequately evaluate *in vivo* tumorigenicity of ahMNCs with or without exogenous genes. U87MG and 578T GBM cells were utilized as positive controls, as GBM is the most prevalent primary tumor in the CNS [20]. U87MG is a human GBM cell line [21, 22], which has been widely used as an *in vitro* and *in vivo* GBM model. 578T GBM cells were primarily cultured from a surgical sample taken from a patient with GBM [23]. Compared with U87MG cells, the 578T cells maintain key features of GBM, such as greater expression of stem cell markers and invasiveness [23]. Moreover, primary culture techniques for 578T cells are similar to those for ahMNCs [7, 23]. The GBM cells were injected into various immune-deficient mice two routes. The recipient immune-deficient mice were adult (4–6 weeks) or neonate (1–2 week old) BALB/c-nu, NOD/SCID, and NOG strains [24, 25]. As a negative control, HBSS media without tumor cells was injection into the brain. Tumor formation was defined as > 20% reduction of body weight plus histological validation in the intracranial (IC) injection model and as ≥ 1,500 mm^3^ of tumor volume plus histological validation in the subcutaneous (SC) injection model [23, 26].

**Fig 1:**
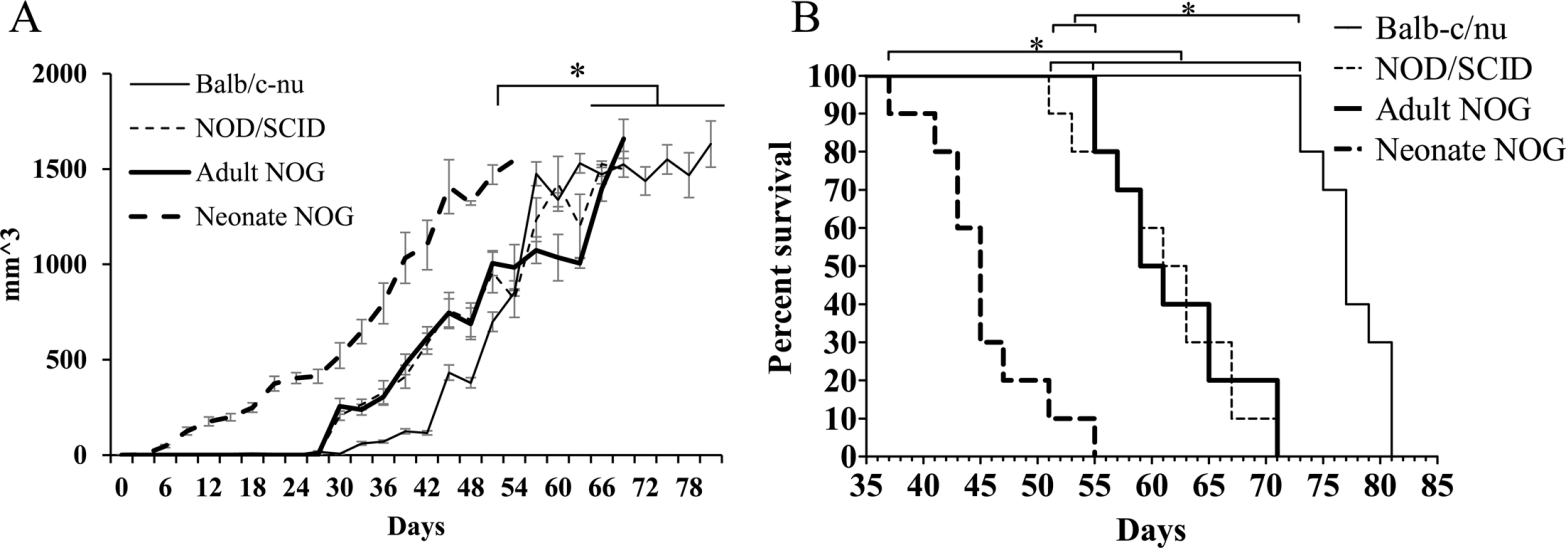
*In vivo* tumor formation of primarily cultured 578T human GBM cells in various animal models. 578T human GBM cells primary cultured from a GBM surgical sample were transplanted into various immune-deficient mouse strains via the subcutaneous (SC) or intracranial (IC) routes. (A) Tumor volume was calculated after 2 × 10^6^ 578T cells were injected into the SC tissue until a volume of 1,500 mm^3^. (B) An aliquot of 2 × 10^5^ cells was stereotactically injected into the brains of mice. Total body weight was monitored, and > 20% total body weight reduction was counted as mortality. *P < 0.05.

*In vivo* tumor formation was significantly faster in neonate NOG mice injected SC with 578T cells (n = 10, median, 54 days) compared with that of adult Balb/c-nu (n = 5, 80 days), adult NOD/SCID (n = 10, 68 days), and adult NOG mice (n = 10, 70 days) (**Fig 1A** and **Table 1**). No difference was observed among the adult strains. *In vivo* tumor formation was also significantly faster in neonate NOG mice injected SC with U87MG cells (n = 10, 42 days) compared with that of adult Balb/c-nu (n = 10, 59 days), adult NOD/SCID (n = 10, 57 days), and adult NOG mice (n = 10, 53 days) (**S1A Fig** and **S1 Table**). Concordant with the results from 578T cells, no difference was detected among the adult strains. Taken together, neonate NOG mice injected SC were most sensitive for *in vivo* tumor formation among our experimental mouse strains.

**Table 1:** Comparison of overall median survivals according to strain, age and route.

Mice	Cells	Age	Route ^a^	Cell No.	Cell form	Event ^b^	Number of mice	Median survivals ^c^
Balb/c-nu	578T	6 weeks	I.C.	2×10^5^	w/ HBSS	Debility	10	77
Balb/c-nu	578T	6 weeks	S.C.	2×10^6^	w/ HBSS	Neoplasm	10	80
NOD/SCID	578T	6 weeks	I.C.	2×10^5^	w/ HBSS	Debility	10	62
NOD/SCID	578T	6 weeks	S.C.	2×10^6^	w/ HBSS	Neoplasm	10	68
Neonate NOG	578T	1 week	I.C.	2×10^5^	w/ HBSS	Debility	10	45
Neonate NOG	578T	1 week	S.C.	2×10^6^	w/ HBSS	Neoplasm	10	54
Adult NOG	578T	6 weeks	I.C.	2×10^5^	w/ HBSS	Debility	10	60
Adult NOG	578T	6 weeks	S.C.	2×10^6^	w/ HBSS	Neoplasm	10	70

a. I.C.: Intracranial injection, S.C.: Subcutaneous injection

b. None: Not happened anything, Debility: decrease of weight and bad condition, Neoplasm: neoplasm into injection site

c. I.C. survivals: Condition and body weight, S.C. survivals: tumor volume ≥ 1,500 mm^3^

*In vivo* tumor formation of neonate NOG mice injected IC with 578T cells (n = 10, 45 days) was significantly faster than that of adult Balb/c-nu (n = 5, 77 days), adult NOD/SCID (n = 10, 62 days), and adult NOG mice (n = 10, 60 days) (**Fig 1B** and **Table 1**). Among adult strains, NOD/SCID and NOG mice showed significantly faster *in vivo* tumor formation than that of Balb/c-nu mice. No difference was observed between adult NOD/SCID and adult NOG mice. In accordance with the 578T cell results, *in vivo* tumor formation of U87MG cells in neonate NOG mice injected IC (n = 10, 21 days) was significantly faster than that in adult Balb/c-nu (n = 10, 33 days), adult NOD/SCID (n = 10, 29 days), and adult NOG mice (n = 10, 27 days) (**S1B Fig** and **S1 Table**). No difference was observed between adult Balb/c-nu and adult NOG mice.

**Fig 2:**
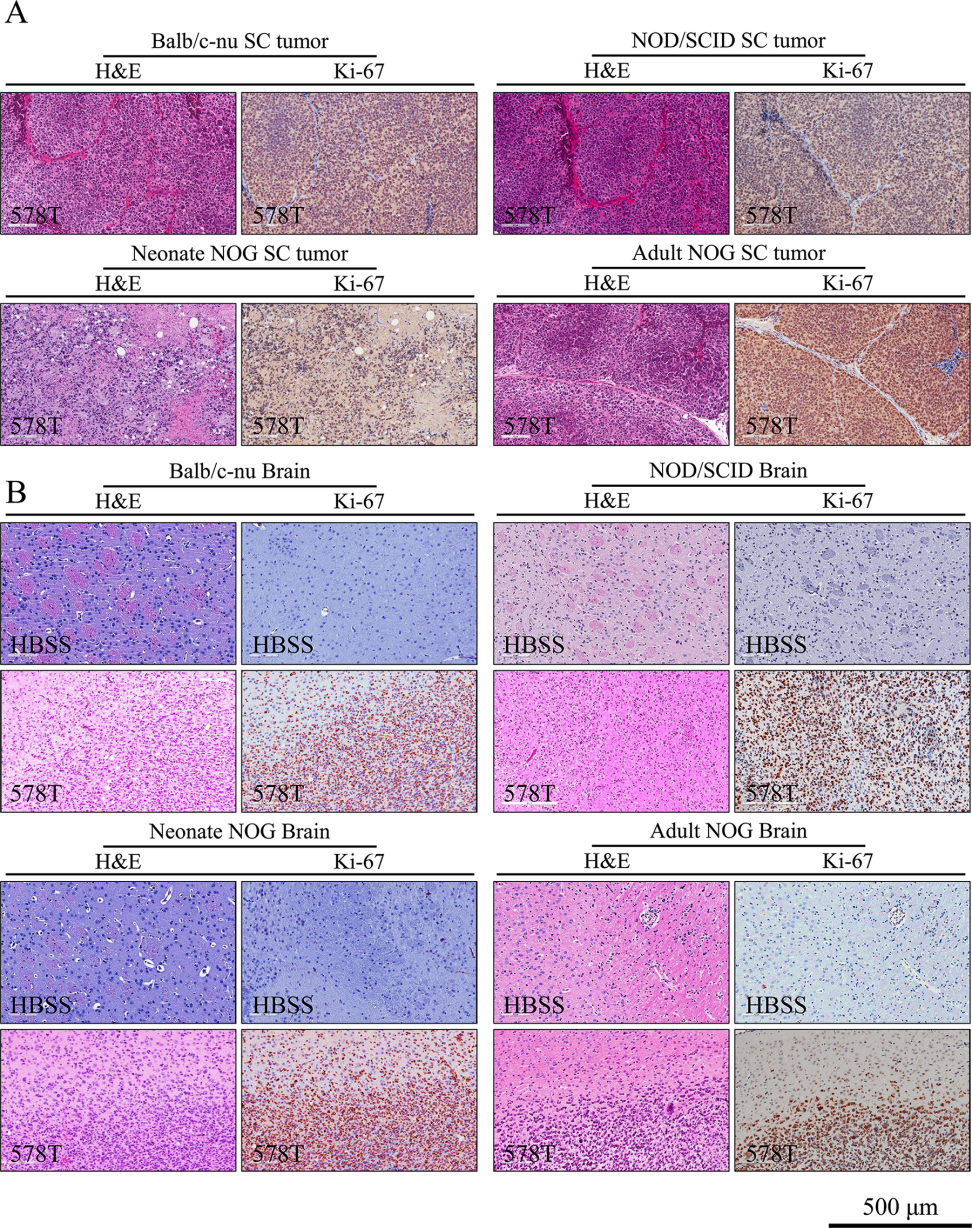
Histological validation of 578T human GBM cell-derived tumors. To validate tumor formation after subcutaneous (A) and intracranial (B) injections, tissue sections were stained with hematoxylin and eosin (H&E) or immunostained against Ki-67, a cell proliferation marker.

In all groups, tumor formation was validated by histological analysis (H&E and Ki-67 immunohistochemistry) [27] (**Fig 2**for 578T and **S2 Fig** for U87MG). In the histological analysis, the negative control mice injected with HBSS did not develop tumors in any case 6 months after the injection (**Fig 2**and **S2 Fig**).

### Immortalization of ahMNCs with hTERT gene transduction

**Fig 3:**
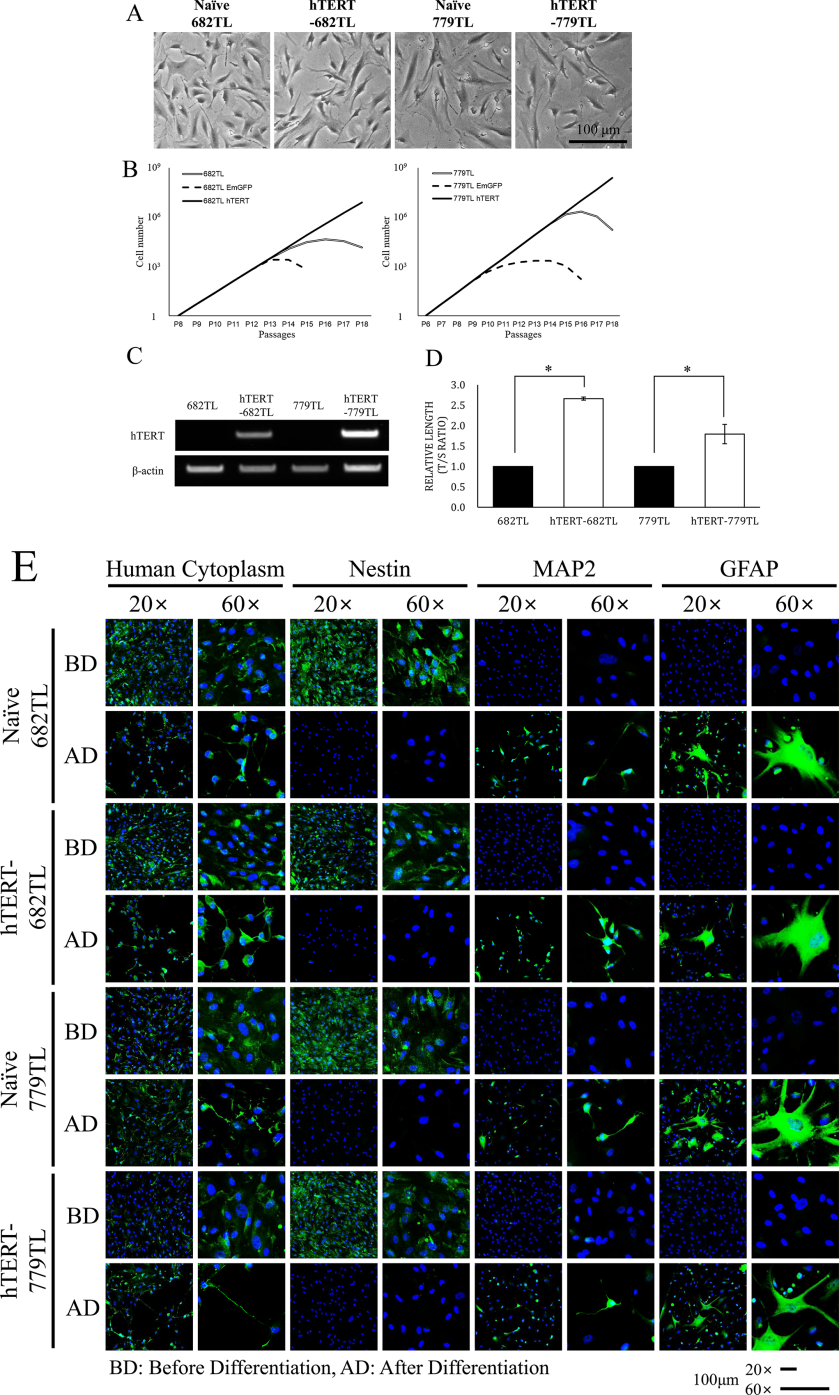
*In vitro* characteristics of ahMNCs and hTERT-ahMNCs. The hTERT gene was transduced into 682TL and 779TL ahMNCs using a lentiviral vector. (A) Morphology of the ahMNCs was compared with that of hTERT-ahMNCs. (B) ahMNCs and hTERT-ahMNCs were cultured *in vitro* for a long time (>18 *in vitro* passages), and the numbers of cells were traced. (C) hTERT expression was confirmed by RT-PCR. (D) Telomere length was analyzed by qPCR and compared. (E) Expression of the neural stem cell marker Nestin and ahMNCs differentiation potential *in vitro* was compared with those of hTERT-ahMNCs. ahMNCs or hTERT-ahMNCs were cultured under differentiating conditions for 12 days. MAP2 for neurons and GFAP for astrocytes. BD; Before differentiation, AD; After differentiation.

To simulate stem cell therapeutics with a therapeutic gene, we introduced the hTERT gene into two kinds of ahMNCs (682TL and 779TL) through lentiviral infection. 682TL and 779TL ahMNCs were primary cultured from temporal lobe surgical samples excised from patients with temporal lobe epilepsy [7]. Infected ahMNCs were selected by blasticidine (5 μg/mL) for 12 days to make colonies. One clone showing continuous proliferation and typical morphology was picked up for each ahMNCs. ahMNCs and ahMNCs with hTERT (hTERT-ahMNCs) had similar morphologies, whereas hTERT-ahMNCs were somewhat elongated (**Fig 3A**). Although there were no significant morphological changes, molecular changes driven by hTERT could be obtained as reported [16]. ahMNCs become senescent after more than 10 passages (P1) [28]. In contrast, hTERT-ahMNCs (hTERT-682TL and hTERT-779TL) proliferate continuously without senescence morphologies (>P18, **Fig 3B**). Stable expression of hTERT in hTERT-ahMNCs was validated (**Fig 3C**). When telomere length was compared, hTERT-ahMNCs showed significantly longer telomeres than those of ahMNCs, as expected (**Fig 3D**). However, expression of stem cell markers and *in vitro* differentiation potential into neural cells did not change by expressing hTERT (**Fig 3E, S4**and **S5 Figs**). Taken together, introducing the hTERT gene using a lentiviral vector could immortalize ahMNCs, but it did not affect stemness or *in vitro* differentiation capacity.

### *In vitro* genetic stability of ahMNCs after hTERT-mediated immortalization

We carried out a karyotype analysis to test the *in vitro* genetic stability of ahMNCs and hTERT-ahMNCs. Karyotypes were interpreted using GTG-banding and Giemsa staining. As shown in **S3 Fig**, long-term cultured ahMNCs (682TL and 779TL at passage 10) showed 2n karyotype without major abnormalities (46 XY and 46 XX, respectively). The hTERT-ahMNCs (hTERT-682TL and hTERT-779TL at passage 18 after clonal selection) also revealed 2n karyotype without major abnormalities (46 XY and 46 XX, respectively). The karyotype analysis was performed two times and each examined 20 cells per each cell type. From the analysis, we could not observe any major abnormalities. These results suggest that the karyotypes of ahMNCs and hTERT-ahMNCs showed no significant chromosomal abnormalities even after long-term *in vitro* expansion or immortalization by lentiviral transduction of *hTERT*.

### *In vitro and in vivo* tumorigenicity of ahMNCs and hTERT-ahMNCs

**Fig 4:**
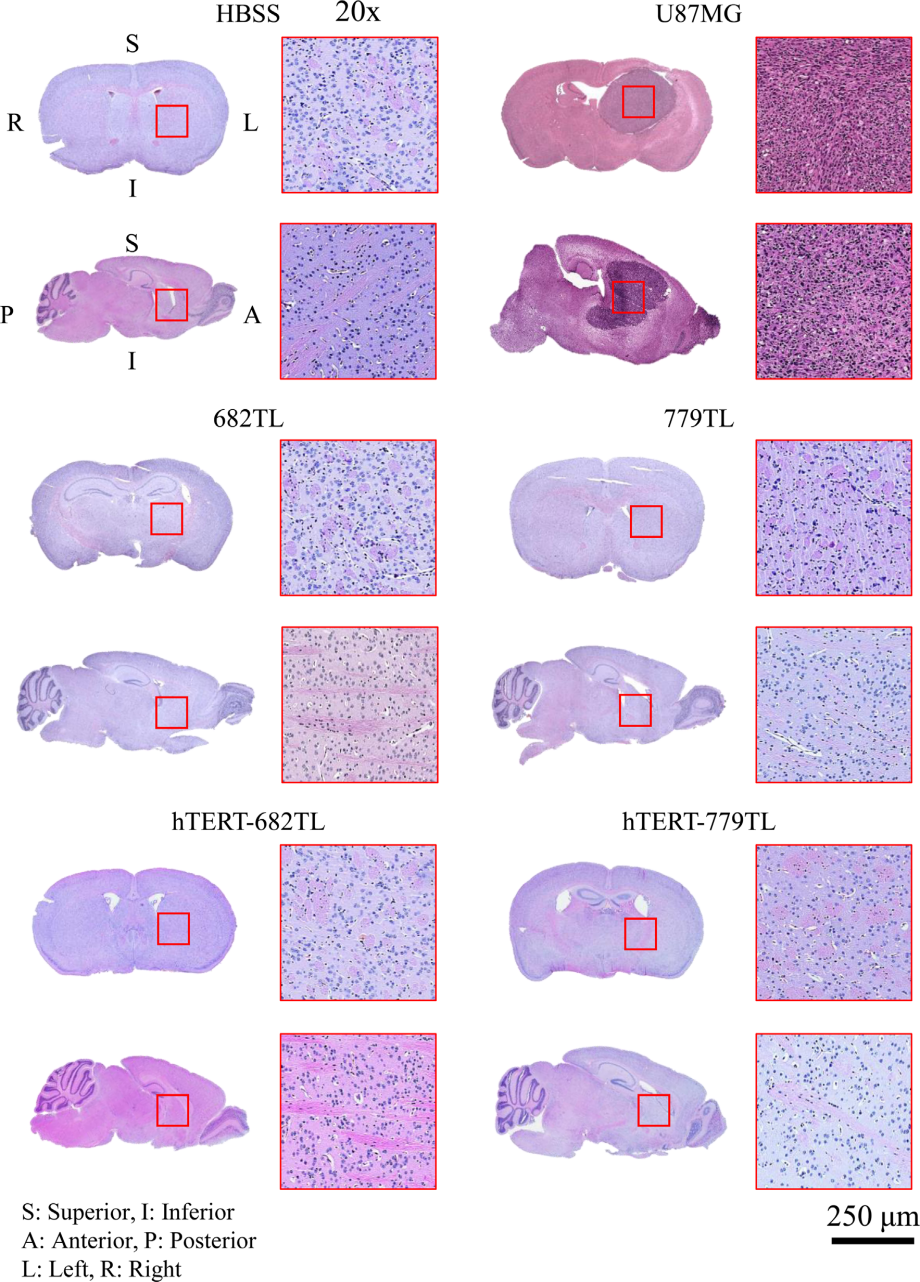
Histology of ahMNCs and hTERT-ahMNCs *in vivo* tumorigenicity. ahMNCs and hTERT-ahMNCs were injected into neonate NOG mice via the intracranial (IC) route. A hematoxylin and eosin (H&E) stained histological analysis showed no tumor formation in the ahMNCs and hTERT-ahMNCs-injected groups 6 months after injection. However, the U87MG injected groups developed brain tumors.

To identify *in vitro* tumorigenicity of ahMNCs and hTERT-ahMNCs, soft agar assay was conducted. We could not observe any proliferating cells from ahMNCs and hTERT-ahMNCs, while U87MG GBM cells made colonies (**S6 Fig**). Next we injected the ahMNCs and hTERT-ahMNCs into immune-deficient mice to test their *in vivo* tumorigenic safety. The cells were injected into neonate NOG mice via the IC or SC route. The positive control U87MG GBM cells resulted in an early decrease of body weight in the IC injected group and tumor masses (≥ 1,500 mm^3^) in the SC injected group, whereas 682TL, hTERT-682TL, 779TL, and hTERT-779TL cells did not induce significant body weight loss or from detectable masses until 6 months after injection (**Table 2**). Tumor formation was confirmed by histological analysis (**Fig 4**). These results suggest that ahMNCs and hTERT-ahMNCs have little *in vivo* tumorigenic potential.

**Table 2:** *In vivo* tumorigenic potential of ahMNCs.

Mice	Cells	Age	Route ^a^	Cell No.	Cell form	Event ^b^	Number of mice	Median Survivals ^c^
Neonate NOG	Negative	1 week	I.C.	-	Only HBSS	None	10	>180
Neonate NOG	Negative	1 week	S.C.	-	Only HBSS	None	10	>180
Neonate NOG	U87MG	1 week	I.C.	2×10^5^	w/ HBSS	Debility	10	21
Neonate NOG	U87MG	1 week	S.C.	2×10^6^	w/ HBSS	Neoplasm	10	42
Neonate NOG	682TL	1 week	I.C.	2×10^5^	w/ HBSS	None	10	>180
Neonate NOG	682TL	1 week	S.C.	2×10^6^	w/ HBSS	None	10	>180
Neonate NOG	hTERT-682TL	1 week	I.C.	2×10^5^	w/ HBSS	None	10	>180
Neonate NOG	hTERT-682TL	1 week	S.C.	2×10^6^	w/ HBSS	None	10	>180
Neonate NOG	779TL	1 week	I.C.	2×10^5^	w/ HBSS	None	10	>180
Neonate NOG	779TL	1 week	S.C.	2×10^6^	w/ HBSS	None	10	>180
Neonate NOG	hTERT-779TL	1 week	I.C.	2×10^5^	w/ HBSS	None	10	>180
Neonate NOG	hTERT-779TL	1 week	S.C.	2×10^6^	w/ HBSS	None	10	>180

a. I.C.: Intracranial injection, S.C.: Subcutaneous injection

b. None: Not happened anything, Debility: decrease of weight and bad condition, Neoplasm: neoplasm into injection site

c. I.C. survivals: Condition and body weight, S.C. survivals: tumor volume ≥ 1,500 mm^3^

## Discussion

Stem cell therapy is an attractive treatment for various neurodegenerative diseases. We reported previously that ahMNCs derived from the temporal lobes of patients with epilepsy helped functional recovery of rats after stroke *in vivo*. However, *in vivo* biosafety must be resolved before clinical application [7, 26]. Traditionally, stem cells are injected into immune-deficient mice to check their tumorigenicity, even though rodent models couldn't represent human completely. Especially, small injection number of stem cells and short observation period due to small body volume and short lifespan of mouse could miss long-term accumulation of adverse chromosomal events in transplanted stem cells which provoke *in vivo* expansion of tumorigenic stem cells and clinically detectable tumors during years or decades of a patient’s post-transplant life.

Among various rodent models, Balb-c/nu mice are commonly used immune-deficient animals for *in vivo* tumorigenicity test of stem cells. However, stem cells with low tumorigenic potential could show a false-negative result within 6 months since Balb-c/nu mice still have a limited immune system, which could affect viability of the stem cells after xenograft. These problems can potentiate the limitation of rodent models in the tumorigenicity test such as small injection cell number and short observation time. We hypothesized that the limitation might be addressed partially by developing a highly sensitive *in vivo* tumorigenicity test. To design a highly sensitive *in vivo* tumorigenicity test, we tried to identify an animal model that provides a good environment for xenograft cells to make tumors. Previously, latent times for tumor formation of U87MG glioma cells in immune-deficient animals of various ages, strains, and injection routes were compared [29]. Among them, the Balb-c/nu strain does not have T cell-dependent immunity. However, they have B cells and natural killer (NK) cells [30]. These immune cells could provoke immune rejection or inflammatory reactions when they meet xenograft human stem cells and prevent tumor formation. The NOD/SCID mouse strain was generated by cross-breeding severe combined immunodeficiency (SCID) mice, which have no T and B cells due to genetic defects [31], with non-obese diabetic (NOD) mice [32]. The NOG mouse strain was developed by eliminating the *Il2rg* gene in the NOD/SCID strain. As NK cells are not produced without *il2rg*, NOG mice are the most prone to tumor formation without significant immune problems *in vivo* [33].

We hypothesized that tumorigenicity could be influenced by strain and age of recipient animals. Neonate mice have been used to test cell tumorigenicity since 1959 [34]. Neonate mice may have a more sensitive biological system than adults to allow carcinogenic responses. The differences might be derived from higher metabolic capacity of developing organs, specific hormone repertoires, or lower immunity [35, 36]. Subsequently, we determined the effects of different transplantation routes on *in vivo* tumor formation. ahMNCs have been transplanted previously via direct IC injection, which mimics the clinical setting of patients suffering from stroke [7]. In this study, we added the SC injection as a comparison. Our tumor formation criteria were > 20% reduction in total body weight after IC injection and ≥ 1,500 mm^3^ tumor volume after the SC injection [26].

As expected, the Balb-c/nu groups showed the latest tumor formation and median survival days, regardless of injection route of either U87MG glioma cells or primary cultured GBM cells (578T). This can be explained by the immune responses to xenogenic cells. However, adult NOD/SCID and NOG mice showed no differences in latent times for tumor formation by either injection route. Neonate NOG mice had the shortest median survival compared with the other age or strain groups. According to our results, neonate NOG mice injected IC could be a suitable model to assess neural stem cell tumorigenicity with or without therapeutic genes.

In the *in vivo* tumorigenic experiments, rates of tumor growth of a GBM line are not quite different from each other in different hosts although detection of tumor initiation is significantly faster in some models. Growth rate and/or proliferation rate of a GBM line in various animal models would be same since same kind (even same batch) of cells was transplanted into them. Regarding that only small population of cancer cells in GBM has tumorigenic potential *in vivo* (i.e., tumor-initiating or cancer stem cells), tumor initiation would represent the ratio of tumor-initiating cells in a cancer cell population [37, 38]. Therefore, faster tumor initiation in certain animal models could represent that the models would detect tumor-initiating cells more sensitively *in vivo*.

The number of cells could also be an important factor affecting safety. We injected 2 × 10^5^ cells via the IC route and 2 × 10^6^ cells SC. These doses are expected to be equal to about 5 × 10^7^ cells for a 60 kg adult [39]. Our dose was 5 times higher than that used in clinical stem cell trials for patients suffering from stroke designed for direct injection of 1 × 10^7^ cells per patient into the brain. We did not observe any tumor formation during the 6 months after either IC or SC high dose injections. Therefore, we could expect that ahMNCs have very low tumorigenic potential *in vivo* at a clinical dose. Nevertheless, smaller injection number should reduce the number of clones transplanted into a rodent animal model, which could under estimate clones with a distinct genomic disruption. In the clinical setting, more distinct clones would be injected into a patient, which increase the risk of tumor-formation. Therefore, tumorigenic potential of ahMNCs should be examined further using primate models, which recapitulate humans better in terms of body volume and lifespan.

Introducing therapeutic genes into stem cells is a promising method to maximize stem cell treatment effects. We introduced the *hTERT* gene into ahMNCs using lentivirus to simulate introducing a therapeutic gene into ahMNCs and examine the effects on ahMNCs tumorigenic potential. As hTERT immortalizes cells, hTERT may have more of an effect on stem cell tumorigenic potential than other possible therapeutic genes [40, 41]. Some reports show tumorigenic potential of hTERT-expressing cells [40], whereas others suggested its safe use for long-term stem cell culture [16]. These results indicate that safety of hTERT should be confirmed. In this study, hTERT overexpression in ahMNCs induced immortalization. However, no tumor formation by ahMNCs with the hTERT gene was detected, even in the most sensitive examination. These results suggest that introducing a therapeutic gene into ahMNCs might be safe in terms of tumorigenic potential, which would increase the clinical implications of ahMNCs.

It was also reported that overexpression of hTERT could result in the change of gene expression patterns of stem cells even after differentiation [16]. Urraca et al. found that neurons derived from permanently hTERT immortalized Dental Pulp Stem Cells (DPSCs) did not show the same gene expression patterns as non-immortalized neurons from wild type DPSCs [16]. The results would indicate that, although hTERT-ahMNCs showed normal karyotypes and minimal tumorigenic potential, it should not guarantee that there are no changes of basal gene expression patterns. Therefore, hTERT might need to be turned OFF during the differentiation/therapeutic stage after expanding ahMNCs *in vitro*.

Genotoxicity is a toxic effect on genetic material that can cause genomic instability and heritable mutations, which often result in transformation. Several methods have been applied at various genetic scales to detect genotoxicity. Traditional G-band karyotyping to detect gross chromosomal abnormalities is well-established and widely used. We did not detect any significant karyotypic abnormalities in parental ahMNCs or hTERT-ahMNCs. However, karyotyping has limited resolution, and can only detect genomic changes >3 Mb in size [42] which implies the difficulties to distinguish abnormalities in gene level, minute chromosomal variations, and mosaicisms with very low abnormal chromosomes. To overcome this limitation, other higher resolution methods have been developed, such as array-based comparative genomic hybridization [43] and single-nucleotide polymorphism arrays [44–46]. More recent sequencing-based methods [47, 48] have the highest resolution to analyze changes in single bases and subgenomic CNVs. It is expected that next generation sequencing will be commonly applied to detect genomic abnormalities in stem cell products in the near future.

Preclinical safety and efficacy test of stem cell products have been designed and conducted using animal models, which could reflect human biology and diseases [49, 50]. Murine models are the mostly widely used *in vivo* safety or efficacy test of stem cells. Although murine models can provide proof-of-concept and mechanism, the physiological properties of them are substantially different from the human, especially immune system [51]. In the case of immune compromised murine models, it is difficult to predict the behavior of stem cells in human body. Compared to murine models non-human primates have similar physiological properties to the human, which include body size, life-span, genetics, and even human cytokines and antibodies. If we can overcome ethical and economic issues, non-human primates will be the best option because they provide more accurate expectation for a clinical application of stem cells.

Biosafety issues should be solved for clinical applications of stem cells. Among them, tumorigenicity is one of the most important issues, as stem cells are expected to exist in the recipients for a long time. Moreover, stem cells have greater tumorigenic potential than that of somatic cells. Therefore, stem cell products should be tested rigorously *in vitro* and *in vivo* before clinical trials. Tests for tumorigenicity remain limited, even though several governments have their own regulations, guidelines, and experimental protocols. In this study, we compared several protocols and found that NOG neonates could be very sensitive animals to test stem cell tumorigenicity. ahMNCs and ahMNCs immortalized by introducing the hTERT gene showed no tumorigenicity using this sensitive platform. Although the use of NOG or NSG mice for tumorigenicity testing is not novel, the sensitivities of various types of immune-deficient mice have not been prepared previously, especially for ahMNCs (one of neural lines). Moreover, this article showed the usefulness of neonatal NOG mice based on their high sensitivity to detect tumor formation of glioblastoma cells, for the first time. These data will be helpful to prepare guidelines to develop stem cell therapeutics and suggest that ahMNCs could be safe to apply to patients with neurodegenerative diseases.

## Supporting Information

S1 Fig *In vivo* tumor formation of U87MG human GBM cells in various animal models. Human U87MG GBM cells were transplanted into various immune-deficient mouse strains via subcutaneous (SC) or intracranial (IC) routes. (A) Tumor volume was calculated after 2 × 10^6^ U87MG cells were injected SC until volume reached 1,500 mm^3^. (B) An aliquot of 2 × 10^5^ cells were stereotactically injected into the brain of mice. Total body weight was monitored, and > 20% total body weight reduction was counted as mortality. * P < 0.05.
(TIF)

S2 Fig Histological validation of U87MG human GBM cell-derived tumors. To validate tumor formation after subcutaneous (A) and intracranial (B) injections of cells, tissue sections were stained with hematoxylin and eosin (H&E) or immunostained against Ki-67, a marker of proliferating cells.
(TIF)

S3 Fig Karyotypic analysis of ahMNCs and hTERT-ahMNCs. Karyotype analysis of ahMNCs and hTERT-ahMNCs was conducted using the G-band method. 682TL and 779TL showed normal 46, XY and normal 46, XX karyotypes, respectively. hTERT-682TL and hTERT-779TL also had 46, XY and normal 46, XX karyotypes, respectively.
(TIF)

S4 Fig FACS analysis of ahMNCs and hTERT-ahMNCs. ahMNCs and hTERT-ahMNCs were characterized by FACS analysis. Nestin, a stem cell marker; MAP2 and Tuj1, markers for neuron; GFAP, an astrocyte marker, and O4, an oligodendrocyte marker.
(TIF)

S5 Fig Quantification of differentiated ahMNCs and hTERT-ahMNCs. ahMNCs (682TL and 779TL) and hTERT-ahMNCs (hTERT-682TL and hTERT-779TL) were cultured under differentiation medium. Nestin-, MAP2-, and GFAP-positive cells were counted before and after differentiation.
(TIF)

S6 Fig *In vitro* soft agar assay of ahMNCs and hTERT-ahMNCs. U87MG, ahMNCs (682TL and 779TL), and hTERT-ahMNCs (hTERT-682TL and hTERT-779TL) were cultured in anchorage-independent culture conditions for 12 days. U87MG showed high sphere formation capacity. However, most of ahMNCs and hTERT-ahMNCs could not survive.
(TIF)

S1 Table *In vivo* tumorigenic potential of U87MG GBM cells in various strain of mice. (TIF)

## References

[1] Lunn JS, Sakowski SA, Hur J, Feldman EL. Stem cell technology for neurodegenerative diseases. Annals of neurology. 2011;70(3):353–61. 10.1002/ana.22487 21905078 PMC3177143

[2] Lindvall O, Kokaia Z. Stem cells in human neurodegenerative disorders—time for clinical translation? J Clin Invest. 2010;120(1):29–40. 10.1172/JCI40543 20051634 PMC2798697

[3] Martino G, Pluchino S, Bonfanti L, Schwartz M. Brain regeneration in physiology and pathology: the immune signature driving therapeutic plasticity of neural stem cells. Physiol Rev. 2011;91(4):1281–304. 10.1152/physrev.00032.2010 22013212 PMC3552310

[4] Fuentealba LC, Rompani SB, Parraguez JI, Obernier K, Romero R, Cepko CL, et al Embryonic Origin of Postnatal Neural Stem Cells. Cell. 2015;161(7):1644–55. 10.1016/j.cell.2015.05.041 26091041 PMC4475276

[5] Drost J, van Jaarsveld RH, Ponsioen B, Zimberlin C, van Boxtel R, Buijs A, et al Sequential cancer mutations in cultured human intestinal stem cells. Nature. 2015;521(7550):43–7. 10.1038/nature14415 . 25924068

[6] Worthley DL, Churchill M, Compton JT, Tailor Y, Rao M, Si Y, et al Gremlin 1 identifies a skeletal stem cell with bone, cartilage, and reticular stromal potential. Cell. 2015;160(1–2):269–84. 10.1016/j.cell.2014.11.042 25594183 PMC4436082

[7] Joo KM, Kang BG, Yeon JY, Cho YJ, An JY, Song HS, et al Experimental and clinical factors influencing long-term stable in vitro expansion of multipotent neural cells from human adult temporal lobes. Experimental neurology. 2013;240:168–77. 10.1016/j.expneurol.2012.11.021 . 23201097

[8] Nombela-Arrieta C, Ritz J, Silberstein LE. The elusive nature and function of mesenchymal stem cells. Nat Rev Mol Cell Biol. 2011;12(2):126–31. 10.1038/nrm3049 21253000 PMC3346289

[9] Nam H, Lee KH, Nam DH, Joo KM. Adult human neural stem cell therapeutics: Current developmental status and prospect. World journal of stem cells. 2015;7(1):126–36. 10.4252/wjsc.v7.i1.126 25621112 PMC4300923

[10] Baraniak PR, McDevitt TC. Stem cell paracrine actions and tissue regeneration. Regen Med. 2010;5(1):121–43. 10.2217/rme.09.74 20017699 PMC2833273

[11] Lee HJ, Lim IJ, Lee MC, Kim SU. Human neural stem cells genetically modified to overexpress brain-derived neurotrophic factor promote functional recovery and neuroprotection in a mouse stroke model. J Neurosci Res. 2010;88(15):3282–94. 10.1002/jnr.22474 . 20818776

[12] Chen B, Gao XQ, Yang CX, Tan SK, Sun ZL, Yan NH, et al Neuroprotective effect of grafting GDNF gene-modified neural stem cells on cerebral ischemia in rats. Brain Res. 2009;1284:1–11. 10.1016/j.brainres.2009.05.100 . 19520066

[13] Marei HE, Farag A, Althani A, Afifi N, Abd-Elmaksoud A, Lashen S, et al Human olfactory bulb neural stem cells expressing hNGF restore cognitive deficit in Alzheimer's disease rat model. Journal of cellular physiology. 2015;230(1):116–30. 10.1002/jcp.24688 . 24911171

[14] Lu P, Jones LL, Snyder EY, Tuszynski MH. Neural stem cells constitutively secrete neurotrophic factors and promote extensive host axonal growth after spinal cord injury. Experimental neurology. 2003;181(2):115–29. . 12781986 10.1016/s0014-4886(03)00037-2

[15] Reddel RR. The role of senescence and immortalization in carcinogenesis. Carcinogenesis. 2000;21(3):477–84. . 10688868 10.1093/carcin/21.3.477

[16] Urraca N, Memon R, El-Iyachi I, Goorha S, Valdez C, Tran QT, et al Characterization of neurons from immortalized dental pulp stem cells for the study of neurogenetic disorders. Stem Cell Res. 2015;15(3):722–30. 10.1016/j.scr.2015.11.004 26599327 PMC4698085

[17] Wilson R, Urraca N, Skobowiat C, Hope KA, Miravalle L, Chamberlin R, et al Assessment of the Tumorigenic Potential of Spontaneously Immortalized and hTERT-Immortalized Cultured Dental Pulp Stem Cells. Stem cells translational medicine. 2015;4(8):905–12. 10.5966/sctm.2014-0196 26032749 PMC4511141

[18] Bocker W, Yin Z, Drosse I, Haasters F, Rossmann O, Wierer M, et al Introducing a single-cell-derived human mesenchymal stem cell line expressing hTERT after lentiviral gene transfer. J Cell Mol Med. 2008;12(4):1347–59. 10.1111/j.1582-4934.2008.00299.x 18318690 PMC3865677

[19] Oh JE, Kim RH, Shin KH, Park NH, Kang MK. DeltaNp63alpha protein triggers epithelial-mesenchymal transition and confers stem cell properties in normal human keratinocytes. The Journal of biological chemistry. 2011;286(44):38757–67. 10.1074/jbc.M111.244939 21880709 PMC3207403

[20] Johnson DR, O'Neill BP. Glioblastoma survival in the United States before and during the temozolomide era. J Neurooncol. 2012;107(2):359–64. 10.1007/s11060-011-0749-4 . 22045118

[21] Clark MJ, Homer N, O'Connor BD, Chen Z, Eskin A, Lee H, et al U87MG decoded: the genomic sequence of a cytogenetically aberrant human cancer cell line. PLoS genetics. 2010;6(1):e1000832 10.1371/journal.pgen.1000832 20126413 PMC2813426

[22] Fogh J, Fogh JM, Orfeo T. One hundred and twenty-seven cultured human tumor cell lines producing tumors in nude mice. Journal of the National Cancer Institute. 1977;59(1):221–6. . 327080 10.1093/jnci/59.1.221

[23] Joo KM, Kim J, Jin J, Kim M, Seol HJ, Muradov J, et al Patient-specific orthotopic glioblastoma xenograft models recapitulate the histopathology and biology of human glioblastomas in situ. Cell reports. 2013;3(1):260–73. 10.1016/j.celrep.2012.12.013 . 23333277

[24] Ishikawa F, Yasukawa M, Lyons B, Yoshida S, Miyamoto T, Yoshimoto G, et al Development of functional human blood and immune systems in NOD/SCID/IL2 receptor {gamma} chain(null) mice. Blood. 2005;106(5):1565–73. 10.1182/blood-2005-02-0516 15920010 PMC1895228

[25] Kanemura H, Go MJ, Shikamura M, Nishishita N, Sakai N, Kamao H, et al Tumorigenicity studies of induced pluripotent stem cell (iPSC)-derived retinal pigment epithelium (RPE) for the treatment of age-related macular degeneration. PloS one. 2014;9(1):e85336 10.1371/journal.pone.0085336 24454843 PMC3891869

[26] Gupta B, Torchilin VP. Monoclonal antibody 2C5-modified doxorubicin-loaded liposomes with significantly enhanced therapeutic activity against intracranial human brain U-87 MG tumor xenografts in nude mice. Cancer immunology, immunotherapy: CII. 2007;56(8):1215–23. 10.1007/s00262-006-0273-0 . 17219149 PMC11030931

[27] Bullwinkel J, Baron-Luhr B, Ludemann A, Wohlenberg C, Gerdes J, Scholzen T. Ki-67 protein is associated with ribosomal RNA transcription in quiescent and proliferating cells. Journal of cellular physiology. 2006;206(3):624–35. 10.1002/jcp.20494 . 16206250

[28] Sakaguchi Y, Sekiya I, Yagishita K, Muneta T. Comparison of human stem cells derived from various mesenchymal tissues: superiority of synovium as a cell source. Arthritis and rheumatism. 2005;52(8):2521–9. 10.1002/art.21212 . 16052568

[29] Kawamata S, Kanemura H, Sakai N, Takahashi M, Go MJ. Design of a Tumorigenicity Test for Induced Pluripotent Stem Cell (iPSC)-Derived Cell Products. J Clin Med. 2015;4(1):159–71. 10.3390/jcm4010159 26237025 PMC4470246

[30] Ganick DJ, Sarnwick RD, Shahidi NT, Manning DD. Inability of intravenously injected monocellular suspensions of human bone marrow to establish in the nude mouse. International archives of allergy and applied immunology. 1980;62(3):330–3. . 6993372 10.1159/000232530

[31] Bosma GC, Custer RP, Bosma MJ. A severe combined immunodeficiency mutation in the mouse. Nature. 1983;301(5900):527–30. . 6823332 10.1038/301527a0

[32] Shultz LD, Schweitzer PA, Christianson SW, Gott B, Schweitzer IB, Tennent B, et al Multiple defects in innate and adaptive immunologic function in NOD/LtSz-scid mice. Journal of immunology. 1995;154(1):180–91. . 7995938

[33] Denton PW, Garcia JV. Humanized mouse models of HIV infection. AIDS reviews. 2011;13(3):135–48. 21799532 PMC3741405

[34] Pietra G, Spencer K, Shubik P. Response of newly born mice to a chemical carcinogen. Nature. 1959;183(4676):1689 . 13666865 10.1038/1831689a0

[35] Flammang TJ, Tungeln LS, Kadlubar FF, Fu PP. Neonatal mouse assay for tumorigenicity: alternative to the chronic rodent bioassay. Regulatory toxicology and pharmacology: RTP. 1997;26(2):230–40. 10.1006/rtph.1997.1125 . 9356286

[36] McClain RM, Keller D, Casciano D, Fu P, MacDonald J, Popp J, et al Neonatal mouse model: review of methods and results. Toxicologic pathology. 2001;29 Suppl:128–37. . 11695548 10.1080/019262301753178537

[37] Kreso A, Dick JE. Evolution of the cancer stem cell model. Cell stem cell. 2014;14(3):275–91. 10.1016/j.stem.2014.02.006 . 24607403

[38] Beck B, Blanpain C. Unravelling cancer stem cell potential. Nature reviews Cancer. 2013;13(10):727–38. 10.1038/nrc3597 . 24060864

[39] Reagan-Shaw S, Nihal M, Ahmad N. Dose translation from animal to human studies revisited. FASEB J. 2008;22(3):659–61. 10.1096/fj.07-9574LSF . 17942826

[40] Hahn WC, Meyerson M. Telomerase activation, cellular immortalization and cancer. Ann Med. 2001;33(2):123–9. 10.3109/07853890109002067 . 11327115

[41] Roy NS, Chandler-Militello D, Lu G, Wang S, Goldman SA. Retrovirally mediated telomerase immortalization of neural progenitor cells. Nature protocols. 2007;2(11):2815–25. 10.1038/nprot.2007.402 . 18007617

[42] Feuk L, Carson AR, Scherer SW. Structural variation in the human genome. Nat Rev Genet. 2006;7(2):85–97. 10.1038/nrg1767 . 16418744

[43] Iafrate AJ, Feuk L, Rivera MN, Listewnik ML, Donahoe PK, Qi Y, et al Detection of large-scale variation in the human genome. Nat Genet. 2004;36(9):949–51. 10.1038/ng1416 . 15286789

[44] Conrad DF, Andrews TD, Carter NP, Hurles ME, Pritchard JK. A high-resolution survey of deletion polymorphism in the human genome. Nat Genet. 2006;38(1):75–81. 10.1038/ng1697 . 16327808

[45] Hinds DA, Kloek AP, Jen M, Chen X, Frazer KA. Common deletions and SNPs are in linkage disequilibrium in the human genome. Nat Genet. 2006;38(1):82–5. 10.1038/ng1695 . 16327809

[46] McCarroll SA, Hadnott TN, Perry GH, Sabeti PC, Zody MC, Barrett JC, et al Common deletion polymorphisms in the human genome. Nat Genet. 2006;38(1):86–92. . 16468122 10.1038/ng1696

[47] Korbel JO, Urban AE, Affourtit JP, Godwin B, Grubert F, Simons JF, et al Paired-end mapping reveals extensive structural variation in the human genome. Science. 2007;318(5849):420–6. 10.1126/science.1149504 17901297 PMC2674581

[48] Tuzun E, Sharp AJ, Bailey JA, Kaul R, Morrison VA, Pertz LM, et al Fine-scale structural variation of the human genome. Nat Genet. 2005;37(7):727–32. 10.1038/ng1562 . 15895083

[49] Frey-Vasconcells J, Whittlesey KJ, Baum E, Feigal EG. Translation of stem cell research: points to consider in designing preclinical animal studies. Stem cells translational medicine. 2012;1(5):353–8. 10.5966/sctm.2012-0018 23197814 PMC3659706

[50] Harding J, Roberts RM, Mirochnitchenko O. Large animal models for stem cell therapy. Stem cell research & therapy. 2013;4(2):23 10.1186/scrt171 23672797 PMC3706788

[51] Goldring CE, Duffy PA, Benvenisty N, Andrews PW, Ben-David U, Eakins R, et al Assessing the safety of stem cell therapeutics. Cell Stem Cell. 2011;8(6):618–28. 10.1016/j.stem.2011.05.012 . 21624806

