# Supplementary figures and images for: Sensitive Tumorigenic Potential Evaluation of Adult Human Multipotent Neural Cells Immortalized by hTERT Gene Transduction
Source: PLoS One. 2016 Jul 8;11(7):e0158639. doi: 10.1371/journal.pone.0158639 (PMC4938125; doi:10.1371/journal.pone.0158639)

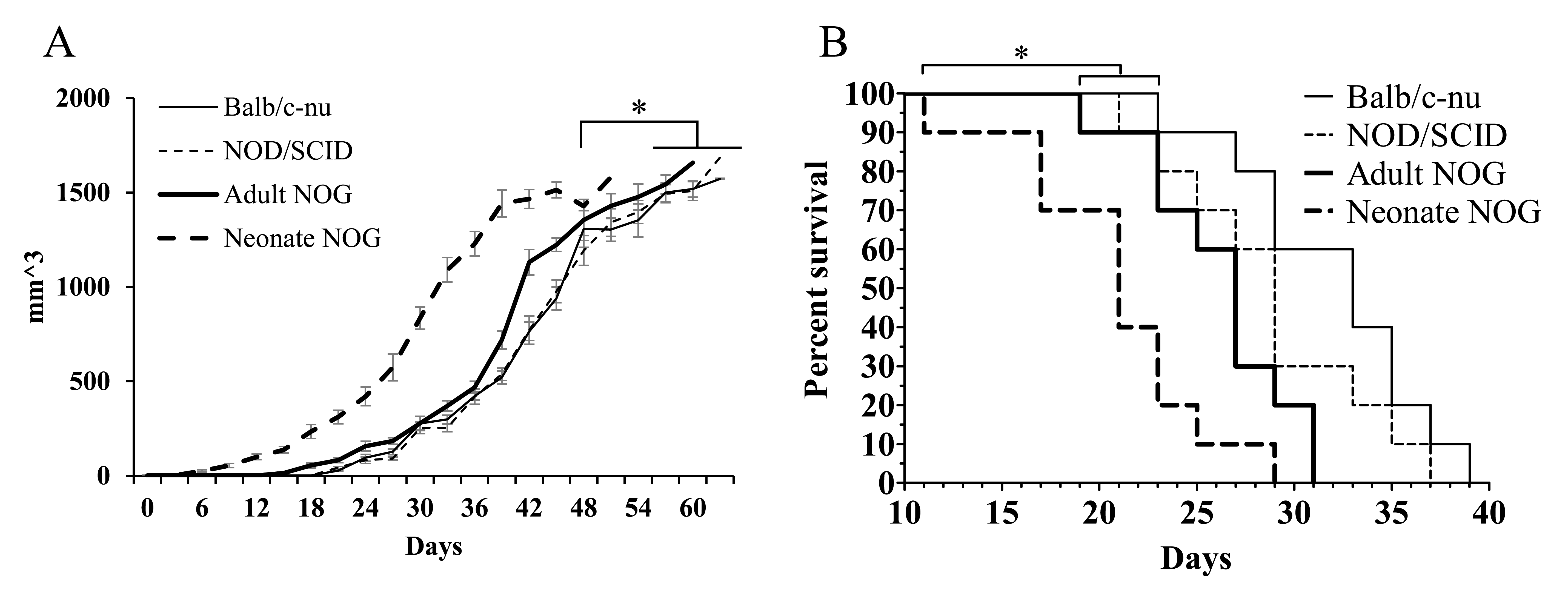

Supplement: S1 Fig — Human U87MG GBM cells were transplanted into various immune-deficient mouse strains via subcutaneous (SC) or intracranial (IC) routes. (A) Tumor volume was calculated after 2 × 10^6^ U87MG cells were injected SC until volume reached 1,500 mm3. (B) An aliquot of 2 × 10^5^ cells were stereotactically injected into the brain of mice. Total body weight was monitored, and > 20% total body weight reduction was counted as mortality. * P < 0.05. (TIF) [file pone.0158639.s001.tif]

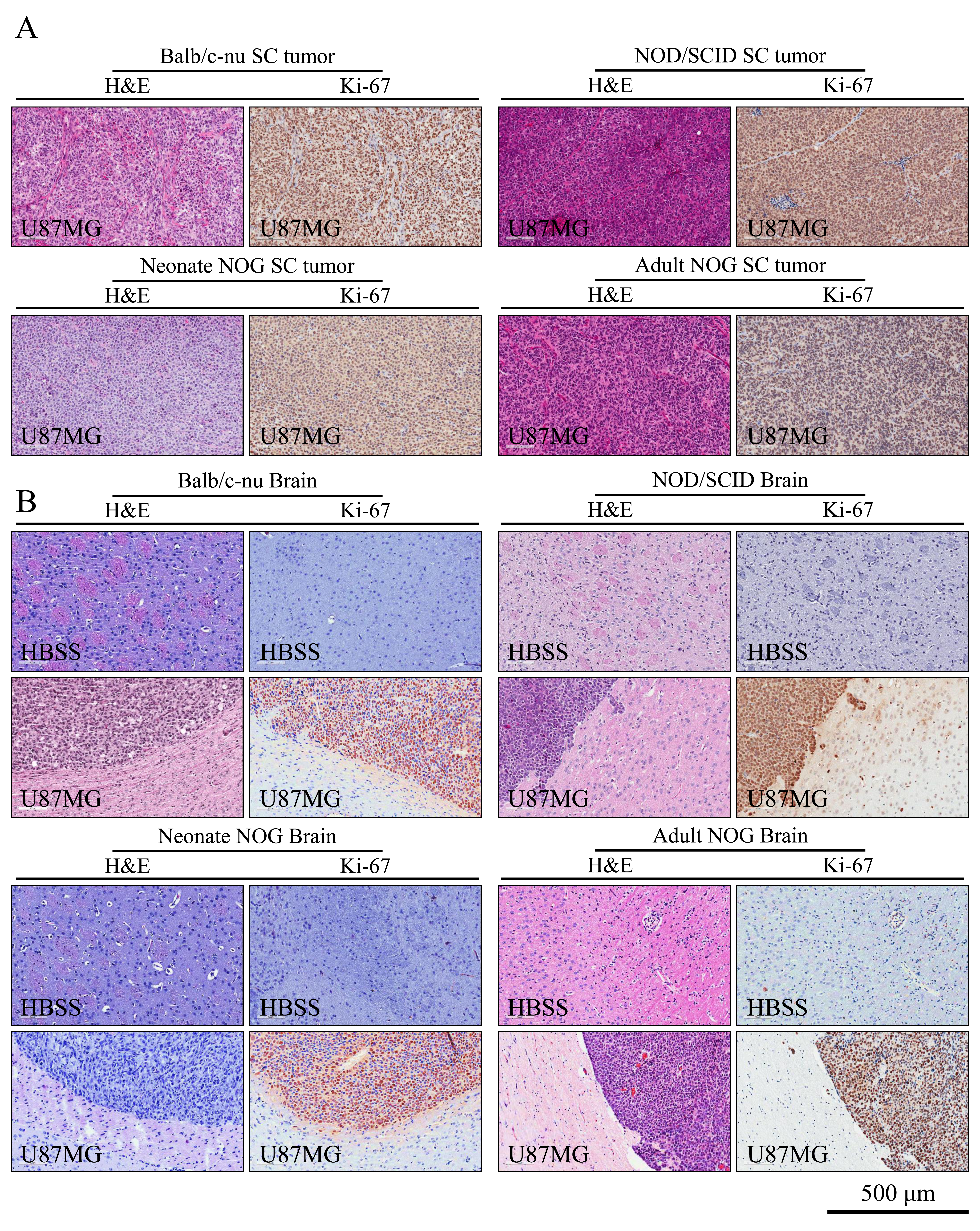

Supplement: S2 Fig — To validate tumor formation after subcutaneous (A) and intracranial (B) injections of cells, tissue sections were stained with hematoxylin and eosin (H&E) or immunostained against Ki-67, a marker of proliferating cells. (TIF) [file pone.0158639.s002.tif]

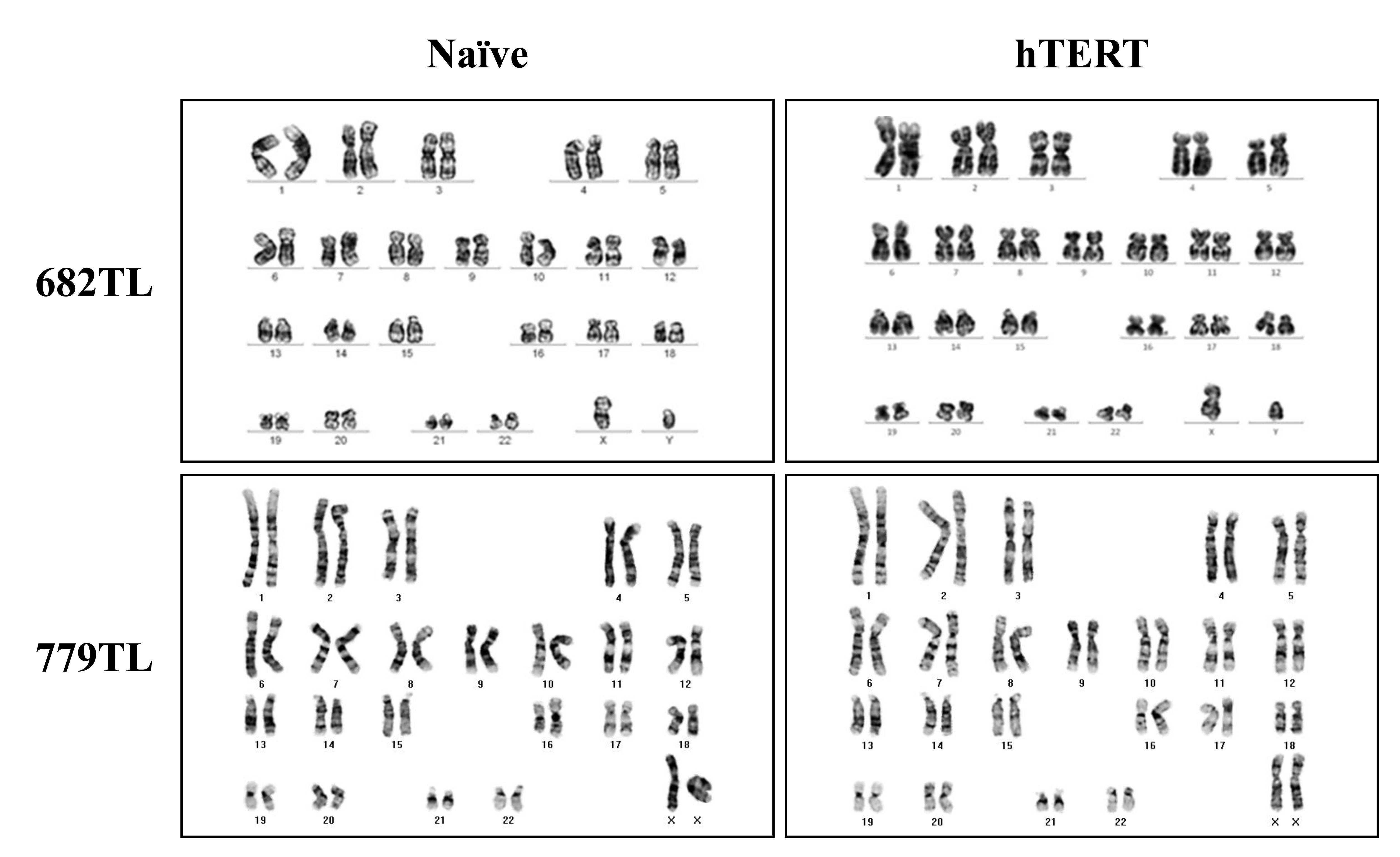

Supplement: S3 Fig — Karyotype analysis of ahMNCs and hTERT-ahMNCs was conducted using the G-band method. 682TL and 779TL showed normal 46, XY and normal 46, XX karyotypes, respectively. hTERT-682TL and hTERT-779TL also had 46, XY and normal 46, XX karyotypes, respectively. (TIF) [file pone.0158639.s003.tif]

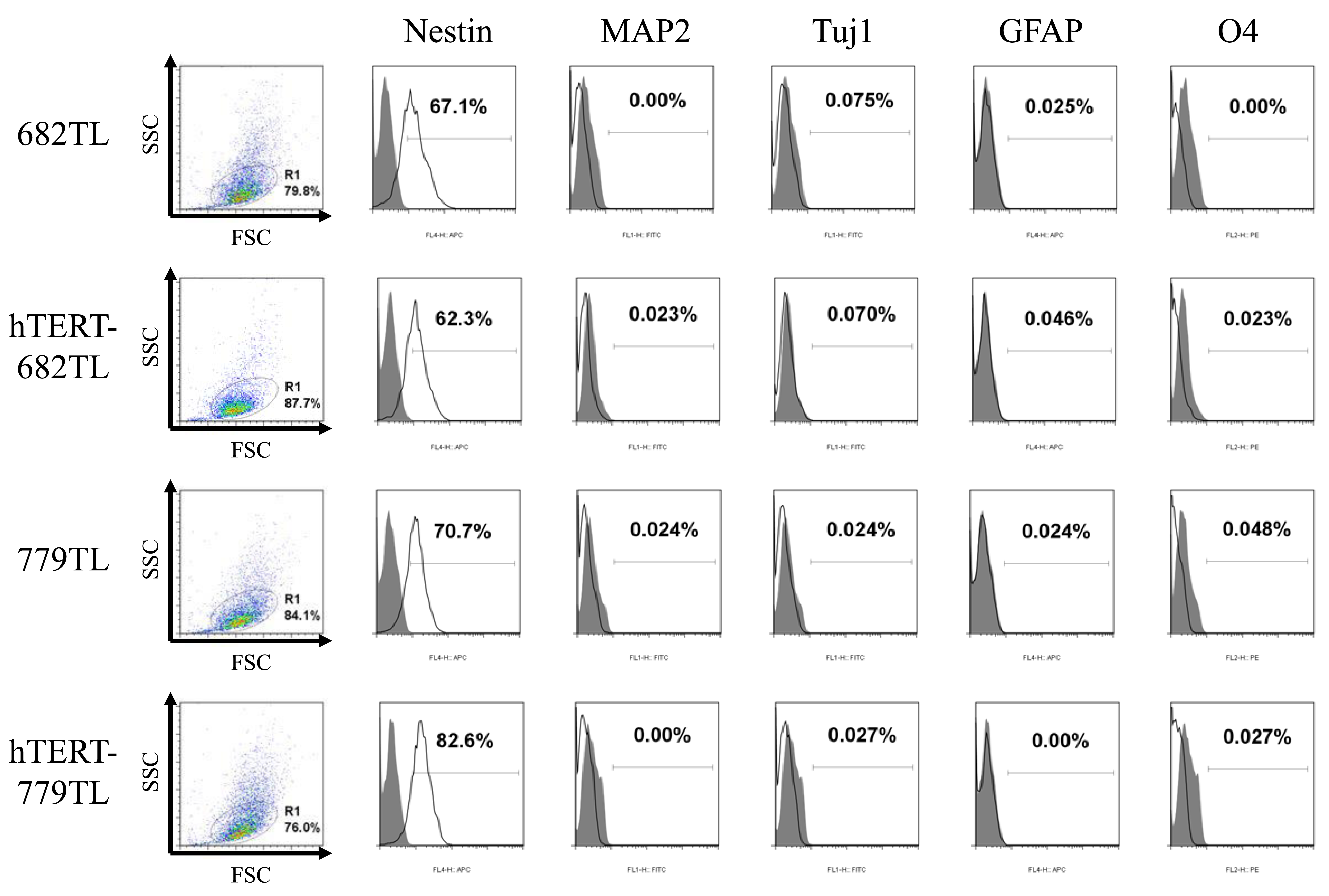

Supplement: S4 Fig — ahMNCs and hTERT-ahMNCs were characterized by FACS analysis. Nestin, a stem cell marker; MAP2 and Tuj1, markers for neuron; GFAP, an astrocyte marker, and O4, an oligodendrocyte marker. (TIF) [file pone.0158639.s004.tif]

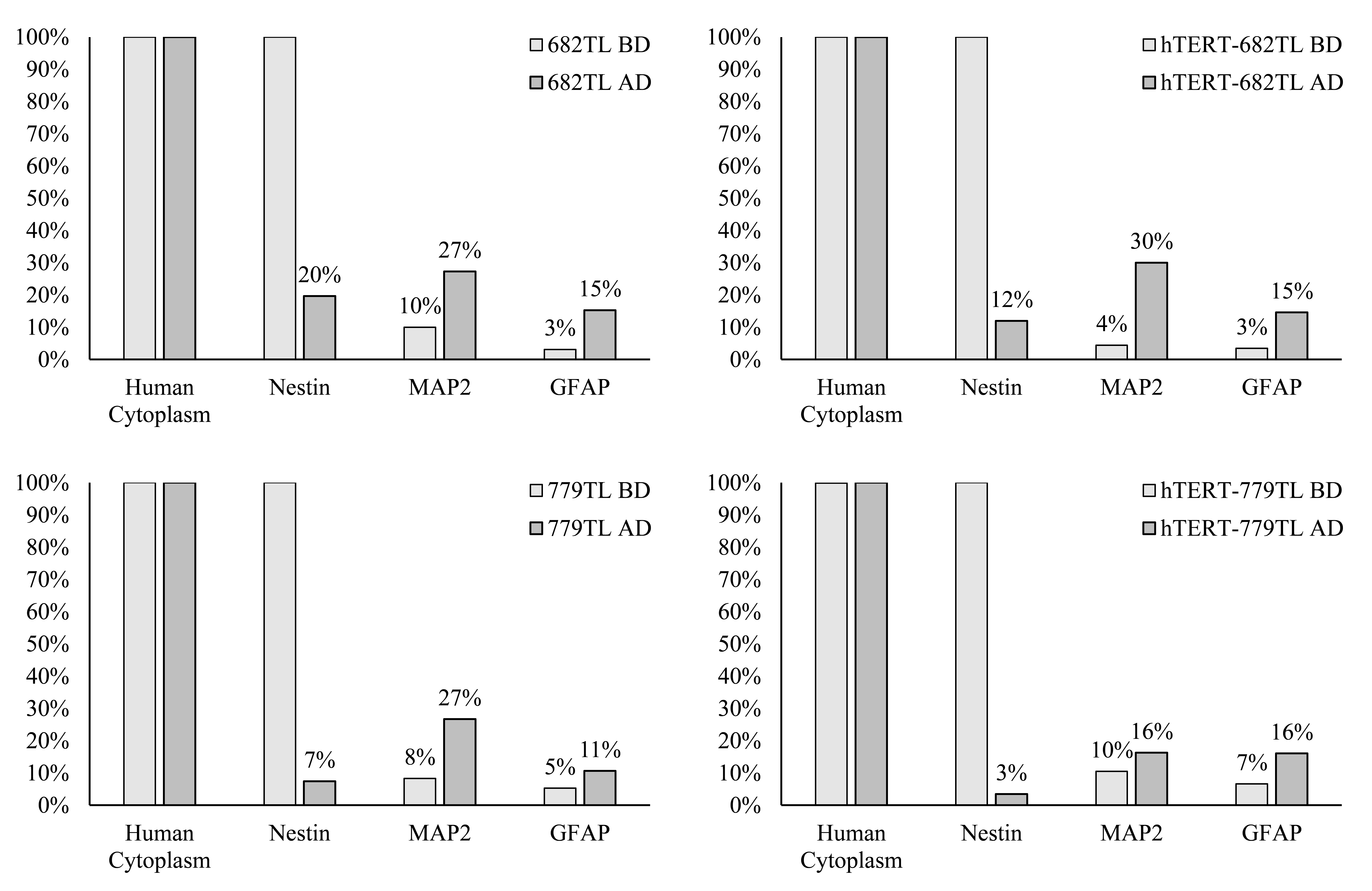

Supplement: S5 Fig — ahMNCs (682TL and 779TL) and hTERT-ahMNCs (hTERT-682TL and hTERT-779TL) were cultured under differentiation medium. Nestin-, MAP2-, and GFAP-positive cells were counted before and after differentiation. (TIF) [file pone.0158639.s005.tif]

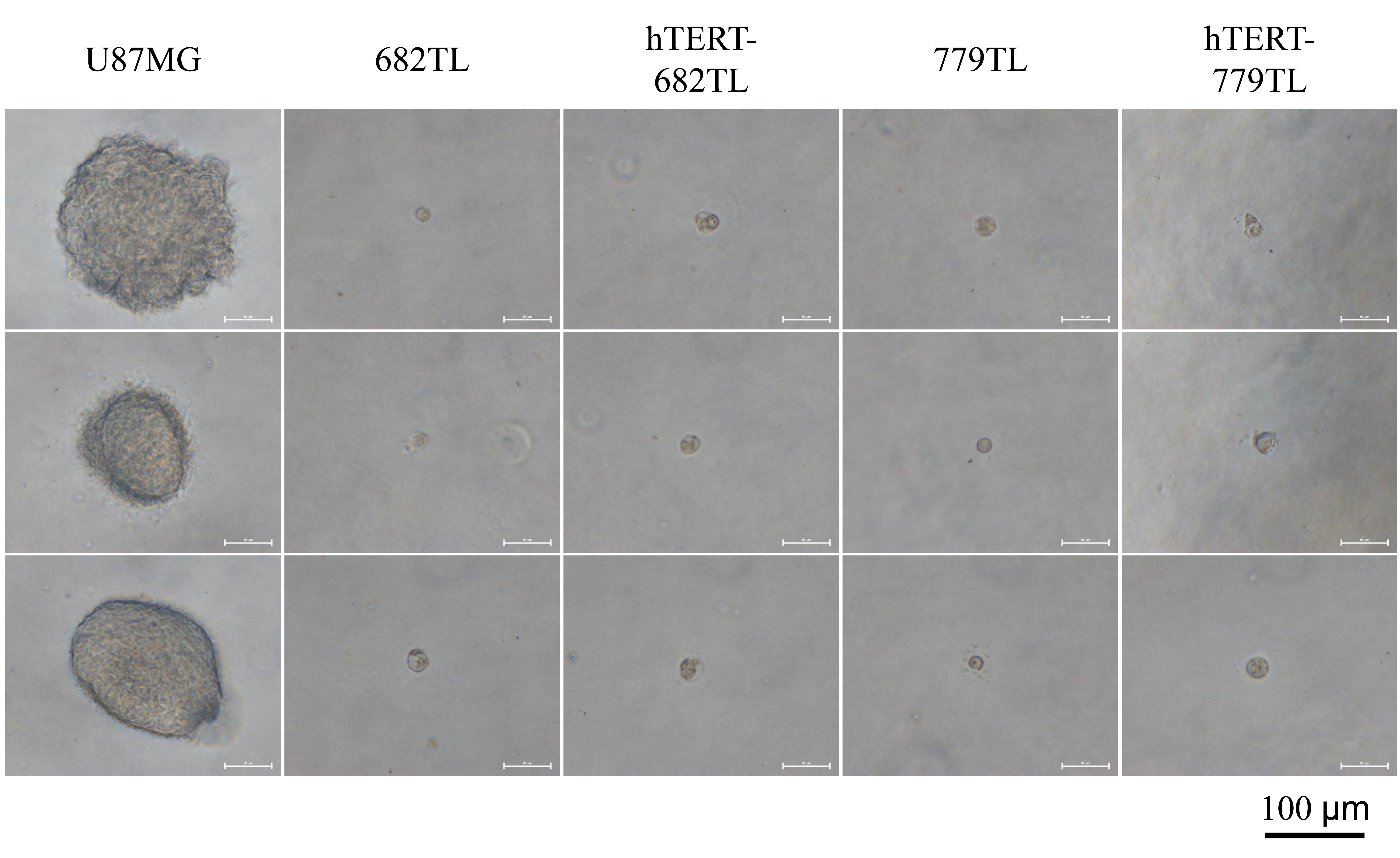

Supplement: S6 Fig — U87MG, ahMNCs (682TL and 779TL), and hTERT-ahMNCs (hTERT-682TL and hTERT-779TL) were cultured in anchorage-independent culture conditions for 12 days. U87MG showed high sphere formation capacity. However, most of ahMNCs and hTERT-ahMNCs could not survive. (TIF) [file pone.0158639.s006.tif]

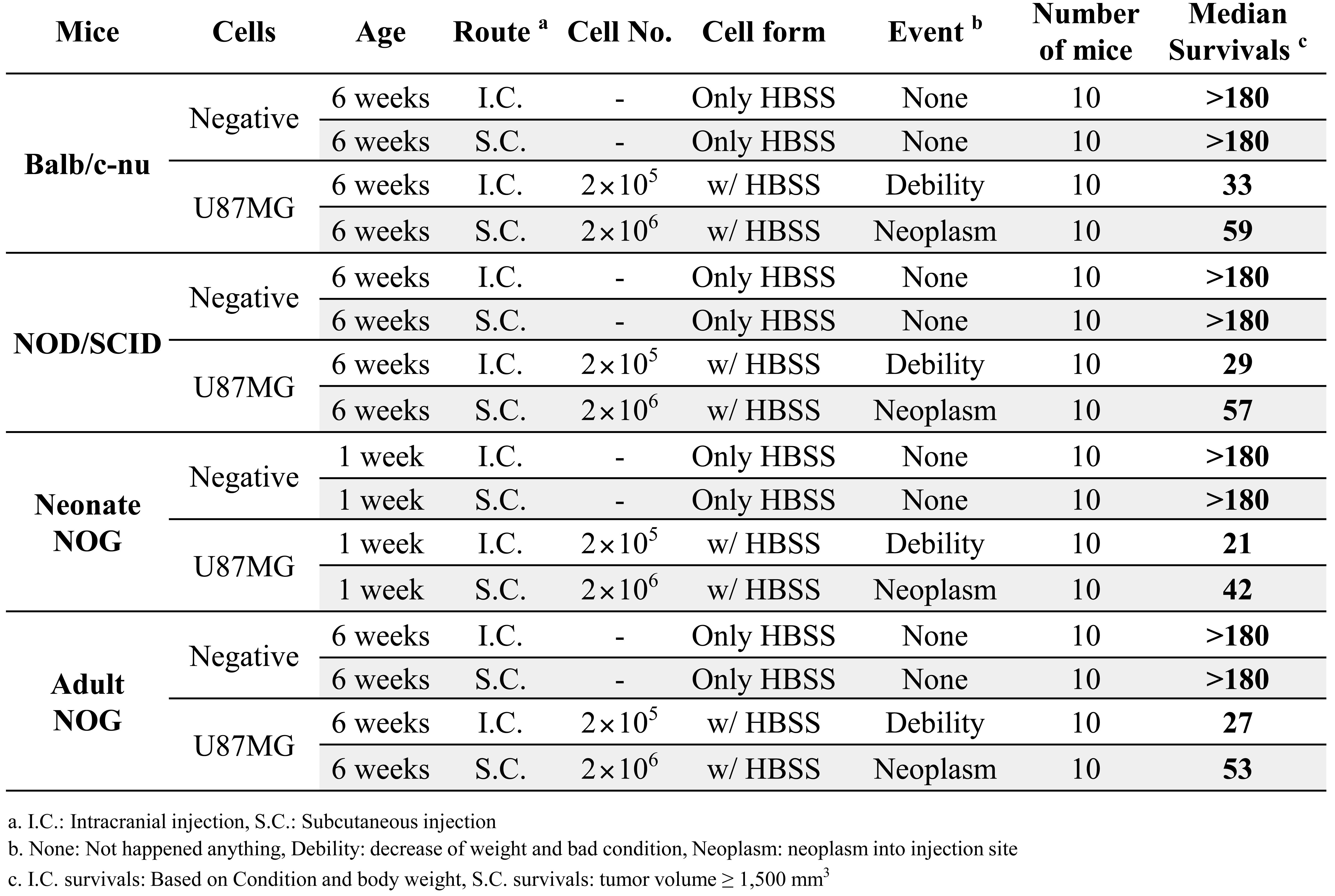

Supplement: S1 Table — (TIF) [file pone.0158639.s007.tif]
